# Applying *tuberculosis management time* to measure the tuberculosis infectious pool at a local level in Ethiopia

**DOI:** 10.1186/s40249-017-0371-6

**Published:** 2017-11-15

**Authors:** Senedu Bekele Gebreegziabher, Gunnar Aksel Bjune, Solomon Abebe Yimer

**Affiliations:** 1 Amhara Regional State Health Bureau, Bahir Dar, Ethiopia; 2 Department of Community Medicine, Institute of Health and Society, University of Oslo, Oslo, Norway; 3 Oslo University Hospital, Oslo, Norway; 4 Department of Bacteriology and Immunology, Division of Infectious Disease Control, Norwegian Institute of Public Health, Oslo, Norway

**Keywords:** Tuberculosis, *TB management time*, Infectious pool, West Gojjam zone, Ethiopia

## Abstract

**Background:**

Measuring the size of the infectious pool of tuberculosis (TB) is essential to understand the burden and monitor trends of TB control program performance. This study applied the concept of *TB management time* to estimate and compare the size of the TB infectious pool between 2009 and 2014 in West Gojjam Zone of Amhara Region, Ethiopia.

**Methods:**

New sputum smear-positive and smear-negative pulmonary TB (PTB) and retreatment cases who attended 30 randomly selected public health facilities in West Gojjam Zone from October 2013 to October 2014 were consecutively enrolled in the study. In order to determine the infectious period, the *TB management time* (number of days from the onset of cough until start of anti-TB treatment) was computed for each patient category. The number of undiagnosed TB cases was estimated and hence the *TB management time* for the undiagnosed category was calculated. The total size of the TB infectious pool during the study period for the study zone was estimated as the annual number of infectious person days.

**Results:**

New smear-positive and smear-negative PTB cases contributed 25,050 and 12,931 infectious person days per year to the TB infectious pool, respectively. The retreatment and presently undiagnosed cases contributed 8840 and 34,310 infectious person days per year, respectively. The total size of the TB infectious pool in West Gojjam Zone during the study period was estimated at 81,131 infectious person days per year or 3405 infectious person days per 100,000 population per year. Compared to a similar study done in 2009 in the study area, the current study showed reduction of the TB infectious pool by 244,279 infectious person days.

**Conclusions:**

*TB management time* is a simple and practical tool that may help to estimate and compare the changes in the size of the TB infectious pool at local level. It may also be used as an indicator to monitor the changes in TB control program performance.

**Electronic supplementary material:**

The online version of this article (10.1186/s40249-017-0371-6) contains supplementary material, which is available to authorized users.

## Multilingual abstracts

Please see Additional file 1 for translations of the abstract into the five official working languages of the United Nations.

## Background

Despite important progress in tuberculosis (TB) control has been made with great global commitment, yet TB remains a major global health problem [1]. According to World Health Organization (WHO) global report, there were an estimated 10.4 million new TB cases and 1.8 million deaths from TB in 2015 [1]. An estimated one-third of the world’s population is infected with TB [2], serving as a reservoir that is continuously contributing to the TB infectious pool.

Measuring the size of the infectious pool of TB is essential to understand the burden and monitor trends of TB control program performance [3]. Currently, WHO uses case notification data, national TB prevalence surveys and data audits to estimate the TB burden [1]. These various sources have yielded considerable information to estimate the TB burden. However, in resource poor countries where problem with data quality such as incomplete records and untimely reports are frequently documented [4], the data from the routine surveillance system may not provide accurate information that shows the real burden of TB. In addition, the national TB prevalence survey which is recommended in high TB burden countries where many cases and deaths are missed by routine reporting are relatively costly and laborious [5, 6]. Currently, there is no simple, inexpensive and practical method that can be applied to measure the TB infectious pool at local level.

A recent study proposed the concept of *TB management time* as an alternative parameter to estimate the TB infectious pool at local level [3]. *TB management time* is basically applied by defining the infectious period contributed by different TB patient categories in a given year. The current study was conducted to achieve two objectives. The first aim was to compare the change in the size of the infectious pool between 2009 and 2014. The concept of *TB management time* was first introduced in 2009 to estimate the infectious pool of TB in West Gojjam Zone of Amhara Region. Therefore, using the same tool we wanted to compare the change in the dynamics of the TB infectious pool between 2009 and 2014. The second aim was that the application of *TB management time* in the previous study had some limitations that needed to be addressed for better application of the tool in a wider perspective. The *TB management time* for smear-negative and retreatment PTB cases were calculated based on evidence obtained from other studies, mostly from low-TB burden countries. Therefore, this study by addressing the limitations of the former study, applied *TB management time* to estimate and compare the size of the TB infectious pool between 2009 and 2014 in the study area.

## Methods

### Study setting

This study was conducted in West Gojjam Zone of Amhara Region, Ethiopia. West Gojjam Zone is one of the ten zones of the Amhara Region. The total population is estimated at 2,382,497 [7]. A total of 30 public health facilities providing TB diagnostic and treatment services were included in the study. Simple random sampling method was used to select study sites. First, we obtained list of all public health facilities providing TB diagnostic and treatment services in West Gojjam Zone. Accordingly, 73 health centers and one hospital were providing TB diagnostic and treatment services during the study period. Of these, 29 health centers were randomly selected. We also added one hospital which is the only available hospital in the study zone. This makes a total of 30 study sites.

Seventy six private health facilities (hospitals and higher clinics) were providing health service to the population in the study zone. Of these, six private health institutions had TB diagnostic and treatment facilities during the study period. However, the private health facilities were not included in this study.

### Operational definition of variables

The national guideline for clinical and programmatic management of TB, which is adapted from the WHO TB treatment guidelines was followed to diagnose, classify and define TB cases [8].

A new case of TB is defined as a patient who has never had treatment for TB or who has taken anti-TB drugs for less than one month.

Smear-positive PTB: a patient with at least two initial sputum smear examinations positive for acid-fast bacilli (AFB) by direct microscopy, or one initial smear examination positive for AFB by direct microscopy and culture positive, or one initial smear examination positive for AFB by direct microscope and radiographic abnormalities consistent with active TB.

Smear-negative PTB: a patient with symptoms suggestive of TB with at least three AFB negative sputum smear examinations, radiographic abnormalities consistent with active PTB, no response to a course of broad spectrum antibiotics and a decision by a clinician to treat with a full course of anti-TB chemotherapy.

Retreatment cases include three sub-categories: treatment failure, relapse and default cases. Treatment after failure is a patient who was started on retreatment after the previous treatment had failed. A default case is defined as a patient who was previously treated for TB and came back for treatment having previously defaulted. A relapse case is a patient who was previously declared cured or treatment completed and is currently diagnosed with bacteriologically positive (sputum smear or culture).

*TB management time* is defined as the time interval from onset of cough until first start of anti-TB treatment.

### Study design, population and data collection

This was a health facility based cross-sectional study conducted in 30 public health facilities in West Gojjam Zone from October 2013 to October 2014. All newly diagnosed smear-positive and smear-negative PTB and retreatment cases ≥15 years of age who attended the study sites during the study period were consecutively interviewed at the time of treatment initiation.

Socio-demographics of patients, symptoms suggestive of TB, date when cough started, date of first visit to health care provider, date of first start of anti-TB treatment were collected using semi-structured questionnaire. The questionnaire was pretested at a health facility for assessing the clarity, consistency and completeness prior to using it for actual data collection. Trained health officers and nurses at each study site collected the data. To assure quality of the data, frequent supervision was made by the principal investigator and other supervisors throughout the data collection period. Extra pulmonary TB cases were not included in this study.

### Data analysis

**Fig. 1:**
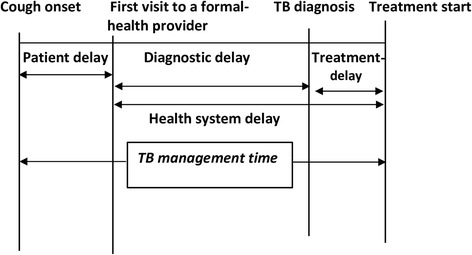
The components of *TB management time.* Note: *TB management time* is defined as the time interval from onset of cough until first start of anti-TB treatment. The figure describes different types of delays within the interval

Data were entered, cleaned and analyzed using IBM Statistical Package for the Social Sciences (SPSS) Version 22 (SPSS Inc. Chicago, IL, USA). Descriptive statistics such as proportions and medians with interquartile ranges (IQRs) were computed. The median *TB management time* for each PTB patient category (new smear-positive, new smear-negative, retreatment and undiagnosed /not yet detected cases) were computed. Figure 1 shows components of *TB management time.*

The median *TB management time* for smear-positive TB patients was calculated based on data collected from new smear-positive PTB cases who attended the study sites during the study period. Previous studies showed that 30% to 58% of smear-positive PTB cases remained infectious after the two weeks of treatment initiation [9, 10]. The median time required for sputum culture conversion after commencement of anti-TB treatment varied from 23 to 39 days for smear-positive cases [11–13]. In order to estimate the total infectious period (the period from onset of cough until the estimated time of non-infectious period following initiation of treatment), an average infectious period of 30 days after the start of treatment was thus added to the median *TB management time* of smear-positive cases.

The median *TB management time* for smear-negative PTB cases was also computed using data obtained from the cross-sectional study conducted in the study area. However, the challenge here was defining the number of smear-negative TB cases that were culture positive. We used data from former local studies to estimate the proportion of culture positive cases from smear-negative cases. The national TB prevalence survey in Ethiopia indicated that 57% of smear-negative cases were culture positive. Another study in Ethiopia revealed that 47% smear-negative cases were culture positive [14, 15]. Based on these facts, we used an average of these two studies (52%) to estimate the number of smear-negative culture positive cases in our study. Finally, by multiplying the calculated number of smear-negative culture positive cases with the calculated median *TB management time,* we estimated the total infectious period for smear-negative TB patients.

The infectious period for retreatment cases was calculated for each retreatment subcategory of patients (relapse, treatment failure and treatment after default). Firstly, the median *TB management time* for relapse, treatment failure and treatment after default cases were computed using data obtained from the cross-sectional study conducted in the study area. For treatment failure cases, the median time period from start of treatment until failed treatment was also computed. Likewise, for treatment after default cases, the median time period from start of treatment until default, and from treatment default until returns to the health facilities and start of retreatment regimen were computed from the data collected in this study.

A recently conducted study in the Amhara Region (study region) showed that the proportion of multi drug resistant TB (MDR-TB) among relapse, treatment failure and default cases were 15.9%, 21.7% and 24.1%, respectively [16]. Previous studies indicated that the median time required for sputum culture conversion for MDR-TB cases varied from 36 days to 90 days [17–19]. However, as the proportion of estimated MDR-TB cases from the retreatment category reported from Amhara Region was not high i.e. within the range of 15.9% – 24.1% [16], we considered an addition of average infectious period of 30 days after the start of retreatment on the calculated *TB management time* for each patient included in the retreatment category.

The undiagnosed TB cases were estimated based on evidence of population-based national TB prevalence survey in Ethiopia [14]. Accordingly, it was estimated that 28% of the smear-positive PTB cases were undiagnosed in the study area. A previous systematic review indicated that undiagnosed PTB cases remain infectious for an average of 3 years [20]. We considered an infectious period of 365 days for the undiagnosed TB cases since our aim was to define the infectious pool for one year.

The total infectious period for new smear-positive and smear-negative PTB and retreatment cases were calculated by multiplying the total number of cases in each patient category during the study period by the total infectious period calculated for each patient category. Likewise, the total infectious period for the undiagnosed cases was calculated by multiplying the total number of estimated undiagnosed cases by the estimated infectious period for this patient category. Finally, the infectious days for each PTB patient category was summed up to estimate the total infectious days contributed by each patient category (smear-positive, smear-negative, retreatment and undiagnosed TB cases) for the study year. The infectious pool was measured in terms of infectious person days per 100,000 population. The total size of the infectious pool of the study zone during the study period was calculated by adding the total number of infectious person days contributed by each TB patient category.

The following assumption and equation that was applied in the former study was used to calculate the total size of TB infectious pool in the current study.

Assumption: Let median infectious period and total number (seen during one year) for smear- positives, smear -negatives, relapse cases, treatment failures, treatment after default cases, and undiagnosed cases be A1 and N1, A2 and N2, A3 and N3, A4 and N4, A5 and N5, A6 and N6 and A7 with N7, respectively. Hence, the estimated total infectious pool can be calculated using the following eq. [3].

$$ \mathrm{Total}\  \mathrm{infectious}\  \mathrm{pool}=\mathrm{A}1\mathrm{Ni}+\dots +\mathrm{A}7\mathrm{N}7+=\sum \limits_{\mathrm{i}}^7\mathrm{AiNi}. $$

## Results

A total of 334 new sputum smear-positive TB cases were included in the study. The median *TB management time* for smear-positive category was 45 days (interquartile range, 23–128 days). By adding an average infectious period of 30 days after commencement of anti-TB treatment, each new smear-positive case was found to have contributed an estimated infectious period of 75 days to the TB infectious pool. A total of 334 new smear-positive TB cases contributed 25,050 infectious person days during the study period (Table 1). In the 2009 study conducted in the same study area, a total of 1250 new smear- positive TB cases contributed 128,750 infectious person days in one year [3].

**Table 1:** Estimated infectious pool of TB in West Gojjam Zone of Amhara Region, Ethiopia from October 2013 to October 2014

Category of TB cases	PTB patients attended at the study sites during the study period (one year)		Infectious period in days	Total estimated infectious person days in a year
	Number	Percent (%)		
New registered PTB cases (*n* = 706)				
Smear-positive	334	47.3	75	25,050
Smear-negative	372	52.7		
Smear-negative culture positive	193^c^	27.3	67	12,931
Retreatment registered cases (*n* = 72)				
Failure	10	13.9	293	2930
Default	8	11.1	192	1536
Relapse	54	75.0	81	4374
Not registered cases				
Undiagnosed cases	94^b^	28.1	365	34,310
Total				^a^ 81,131

^a^Total estimated infectious person days per 100,000 population per year is 3405

^b^We assumed 28% of smear-positive cases were undiagnosed based on the national TB prevalence survey report [14]

^c^Among a cohort of smear-negative cases enrolled in the study, 52% of smear- negative cases were estimated to be culture positive

*TB* tuberculosis

*PTB* Pulmonary tuberculosis

**Fig. 2:**
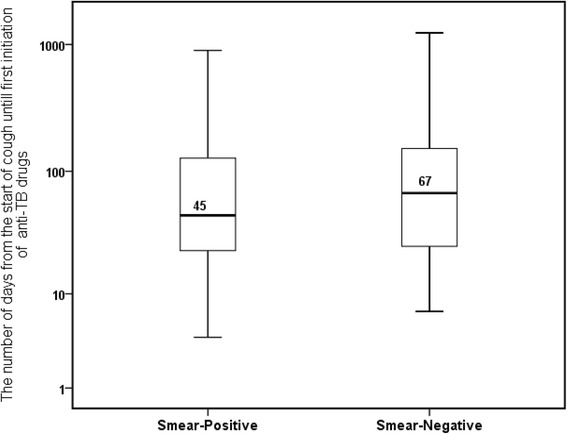
Box plots depict the median *TB management time*. Note: Box plots showing the median *TB management time* from cough until start of TB treatment for new smear-positive and new smear-negative pulmonary TB cases

There were 372 new smear-negative TB cases. Among these, 193 (52%) were estimated to be culture positive cases. The median *TB management time* for new smear-negative patients was 67 days (interquartile range, 25–152 days). A total of 193 smear-negative cases contributed 12,931 infectious person days. The median *TB management time* estimated for new smear-negative PTB cases is high compared to new smear-positive PTB cases (Fig. 2). In 2009, 1998 new smear-negative PTB cases identified in the current study area contributed 39,960 infectious person days to the infectious pool of TB in one year [3].

It was estimated that 94 (28%) of new sputum smear-positive TB cases were undiagnosed in the study zone. The estimated *TB management time* for this category was 365 days, thus 94 undiagnosed TB cases contributed 34,310 infectious person days to the TB infectious pool. In 2009, the estimated 416 undiagnosed cases contributed 151,840 infectious person days to the TB infectious pool in the study area [3].

The median *TB management time* for relapse cases was 51 days (interquartile range 24–97 days). By adding an infectious period of 30 days after start of retreatment, one relapse case contributed 81 infectious person days. A total of 54 relapse cases identified in the study thus contributed 4374 infectious person days. In 2009, 45 relapse cases contributed 2700 infectious person days to the TB infectious pool in the study area [3].

The median *TB management time* for treatment failure cases was 98 days (interquartile range 47–235 days). The median time from start of treatment until failed treatment was 165 days. In addition, an infectious period of 30 days after the commencement of retreatment was added. Accordingly, the infectious period of one treatment failure case was estimated at 293 days. A total of ten treatment failure cases enrolled in our study thus contributed 2930 infectious person days to the TB infectious pool. Whereas in the 2009 study, a total of nine treatment failure cases contributed 1620 infectious person days during a year period [3]. The median *TB management time* for treatment after default cases was 43 days (interquartile range 25–105 days). The median time period from start of treatment until default treatment was 49 days, while the median time period from default treatment until returns to the health facilities and start of retreatment regimen was 70 days. By adding an average infectious period of 30 days after start of retreatment, the contribution of one treatment after default case was estimated at 192 infectious person days. A total of eight treatment after default cases enrolled in this study contributed 1536 infectious person days to the TB infectious pool. Six treatment after default cases contributed 540 infectious person days to the TB infectious pool of the study area in 2009 [3].

The total estimated infectious pool of TB from October 2013 to October 2014 for West Gojjam Zone of Amhara Region was 81,131 person days or 3405 infectious person days per 100,000 population. In contrast, the total infectious pool of TB in the study area was estimated at 325410 person days or 15,447 person days per 100,000 population in the 2009 study [3].

## Discussion

Measuring the size of the infectious pool of TB is essential to understand the burden and monitor trends of TB control program performance [3]. In this study, we estimated the TB infectious pool using *TB management time* as a simple tool. The estimated infectious person days contributed to the TB infectious pool from new smear-positive patients in the current study is lower than that reported from the former study [3]. The main reason for this difference may be related to the significant change in the value of the *TB management time* between the current study (45 days) and the former study that showed, 73 days. This may indicate improvement in health seeking behavior among patients and the diagnostic capacity of health facilities. Secondly, the numbers of smear-positive PTB cases treated in one year in the previous study were more than the current study and thus contributed more infectious person days.

It has been shown from low-TB burden countries that smear-negative patients are capable of transmitting the disease [21–24], and new infections originating from them significantly contribute to the burden of TB transmission [25]. The contribution of smear-negative cases to the TB infectious pool in the former study was estimated by applying the evidences from developed countries [3, 26]. Accordingly, smear-negative cases contributed 20% of the smear-positive cases. In the current study however, the contribution of smear-negative culture positive cases were estimated based on evidence from Ethiopia [14, 15], and the result indicates that smear-negative culture positive cases contributed 15.9% of infectious person days to the infectious pool.

The median *TB management time* estimated for new smear-negative PTB cases is high compared to new smear-positive PTB cases. This is due to the fact that majority of health centers of the study area were relying on smear microscopy to diagnose PTB during the study period. Smear microscopy has very low sensitivity [27], and many patients can get false negative results. According to the national TB diagnostic and treatment guideline of Ethiopia [8], the diagnostic process for smear**-**negative TB patients may take between 15 and 30 days before anti**-**TB treatment is initiated. Therefore, this long time duration before diagnosis and start of treatment for smear-negative cases may increase the median *TB management time*.

The undiagnosed TB cases contributed the largest number of infectious person days to the infectious pool. Undiagnosed cases remained infectious throughout the year [20], and serve as a continuous pool for generating new infections. Nevertheless, the estimated infectious person days for this category of patients in the current study is relatively lower compared to the study in 2009 [3]. This may be related to improved TB diagnostic and treatment facilities in the study area [28]. As the health seeking behaviour and diagnostic facilities are improved, the undiagnosed cases may be detected. The geographical DOTS coverage in Ethiopia and the study region is 100% [14, 29], indicating that most TB patients have access to TB diagnosis and treatment services. This may reduce diagnostic delay and the backlog of undiagnosed TB cases. However, the result of the current study should be interpreted cautiously given smear microscopy was the basic diagnostic tool used to diagnose TB during the study period.

The retreatment category accounted for 11% of the total size of the TB infectious pool. This is higher compared to the 2009 study report which was 1.5%. In the current study, the median *TB management time* for the sub categories of the retreatment group were calculated from the data obtained in this study. This may be considered a relatively acceptable estimate. However, the infectious periods for the retreatment subcategories in the 2009 study were computed based on evidences obtained from other studies. In addition, the average infectious period after start of treatment for retreatment cases is updated in the current study.

Estimating the infectious pool of TB requires defining the infectious period for each TB patient category. The beginning of the infectious period is when onset of symptoms occurs, especially cough [30]. Cough is the cardinal symptom of PTB [31]. Ninety eight percent new and all retreatment PTB cases in our study reported cough, and most were able to report the time when their cough first started. We assumed the date of first onset of cough and date of first treatment initiation as important parameters needed to define the *TB management time*. The application of the tool (*TB management time)* can be used and evaluated at the local level. TB control program managers at local can use the tool to analyze changes in the TB infectious pool and monitor the performance of the TB control program.

The size of the infectious pool of TB estimated using *TB management time* in West Gojjam Zone during October 2013 to October 2014 is lower compared to that estimated in 2009 [3]. This may be related to a number of reasons that may reduce delay in TB diagnosis and treatment of TB. Improved access to TB diagnostic and treatment services [28], and the increasing involvement of health extension workers (community health workers) in early identification and referral of TB suspects to the nearest health facilities where AFB smear microscopy test is available are the most likely reasons. Furthermore, the number of patients and infectious period for each patient category plays pivotal role in determining the size of the TB infectious pool. As described earlier, the numbers of cases in each patient category in the current study were lower than the former study [3].

### Limitation of the study

This study has potential limitations that should be considered for improved application of the tool. The study was carried out only in government health facilities. Private health facilities were not included. Therefore, one may argue that number of patients seen in private health facilities may have an effect in the estimation of the size of the infectious pool. However, as the number of private health facilities involved in TB diagnostic and treatment services in the study zone is very low, it may not have a significant effect in the infectious pool estimate. The number of undiagnosed PTB cases during the study period was estimated based on the national TB prevalence survey result in Ethiopia, and may vary across the different regions of the country. This may have resulted in under or over estimation of undiagnosed cases in this study. However, the undiagnosed cases will be identified sooner or later as access to diagnostic and treatment facilities and the health seeking behavior of patients improve. We also believe that the undiagnosed cases are not decisive category for the application of the tool.

In addition, some patients may not accurately remember the exact date of onset of their symptoms and is subject to recall bias. However, a local calendar listing the main religious and national days was used to help patients remember the date of onset of their symptoms.

One of our objectives for conducting the current study was to address some limitations related to using *TB management time*. One may ask the validity of comparing the 2009 infectious pool size with the 2014 while some proportions used in the parameter were adjusted for the current study. The limitations were related to defining the infectious periods for smear-negatives, retreatment and undiagnosed TB cases. In the 2009 study, the infectious period for smear-negatives was calculated considering that smear-negative TB cases contributed 20% of smear-positive cases and this was applied to estimate infectious person days that contributed to infectious pool from smear-negative category. While in the current study, we computed the median *TB management time* for smear-negative cases from the data collected in this study. We also estimated the proportion of smear-negative culture positive cases based on the local evidence [14, 15]. In the previous study, smear-negative category accounted for 12.3% of the infectious pool while in the current study accounted for 15.9% of the size of the infectious pool making a difference of 3.6% between the previous and the current studies. While the undiagnosed TB case proportion used in the 2009 study was estimated at 33%, we used 28% in the current study making a 5% difference between the previous and the current study. As described earlier, the retreatment category in the previous study accounted for 1.5% of the infectious pool while in the current study, it accounted for 11% of the size of TB infectious pool. The size of the infectious pool in 2014 has shown decline when compared with the year 2009. On the other hand, if we had applied similar proportions for smear-negatives, retreatment and undiagnosed TB cases used in 2009 for 2014, the total size of the infectious pool would still have been much lower than that in 2009. This indicates that the adjustments we applied for 2014 does not make a big difference in the total size of the infectious pool between 2009 and 2014. Overall, this demonstrates that the tool can be used to monitor the size of the TB infectious pool in different time periods.

## Conclusions

The total infectious pool of TB estimated using *TB management time* from October 2013 to October 2014 in West Gojjam Zone is lower compared to that estimated in 2009. The undiagnosed TB patient category followed by the smear-positive patient group contributed the largest infectious person days to the infectious pool in the study zone.

A simple and inexpensive tool is essential to estimate the infectious pool of TB and monitor program performance at local level. Systematic recording of *TB management time* in the unit TB registry book may help to estimate the infectious pool of TB and monitor trends of TB control program performance at the local level. Additional validation studies including both public and private health facilities need to be conducted before full-scale implementation of the parameter. In addition, further research is needed to validate the contribution of pulmonary smear-negative and retreatment cases to the infectious pool of TB. Moreover, a study that explores the feasibility of implementing the parameter at local level is warranted.

## Additional files

Additional file 1: Multilingual abstracts in the five official working languages of the United Nations. (PDF 671 kb)

Additional file 2: Data set used for the article. The data set consists of 706 new pulmonary TB cases included in the study and the variables used in this article. (XLS 226 kb)

Additional file 3: Data set used for the article. The data set consists of 72 retreatment pulmonary TB cases included in the study and the variables used in this article. (XLS 53 kb)

## Abbreviations

AFB: acid-fast bacilli
DOTS: directly observed treatment short-course
IQR: interquartile range
MDR-TB: multi-drug resistant TB
PTB: pulmonary tuberculosis
SPSS: statistical package for the social sciences
TB: tuberculosis
WHO: World Health Organization
