# Supplementary material for: Applying tuberculosis management time to measure the tuberculosis infectious pool at a local level in Ethiopia
Source: Infect Dis Poverty. 2017 Nov 15;6:156. doi: 10.1186/s40249-017-0371-6 (PMC5686949; doi:10.1186/s40249-017-0371-6)
Supplement: Supplementary file 1 — Multilingual abstracts in the five official working languages of the United Nations. (PDF 671 kb) [file 40249_2017_371_MOESM1_ESM.pdf]

Translation of the abstract into the five official working languages of the United Nations

تطبيق زمن *إدارة مرض السل* لقياس تجميعة السل المُعدية على المستوى المحلي في إثيوبيا

سينيدو بيكيل جبريغزيابر وغونار أكسل بجون وسولومون أبيبي ييمر

ملخص
خلفية: <bx/> إن قياس حجم التجميعة المعدية ل مرض السل (TB) أمر ضروري لفهم العبء ورصد اتجاهات أداء برنامج مكافحة السل. طبقت هذه الدراسة مفهوم زمن إدارة مرض السل لتقدير ومقارنة حجم تجميعة السل المعدية بين عامي 2009 و 2014 في إقليم غرب غوجام بمنطقة أمهارا، إثيوبيا.
الوسائل: تم إدراج حالات جديدة من حالات السل الرئوي (PTB) إيجابي اللطاخة البلغمية وسلبي اللطاخة وحالات إعادة العلاج، الذين زاروا 30 من مرافق الصحة العامة المختارة عشوائيًا في منطقة غرب غوجام من أكتوبر 2013 إلى أكتوبر 2014، تباعًا في الدراسة. لتحديد فترة العدوى، تم حساب زمن إدارة مرض السل (عدد الأيام منذ بداية السعال وحتى بداية العلاج بمضادات السل) لكل فئة من فئات المرضى. تم تقدير عدد حالات السل غير المشخصة، ومن ثم حساب زمن إدارة مرض السل للفئة غير المشخصة. وقُدِّر الحجم الكلي لتجميعة السل المعدية خلال فترة الدراسة للمنطقة الخاضعة للدراسة، أنها العدد السنوي لأيام الشخص الناقل للعدوى.
النتائج: ساهمت الحالات الجديدة للإصابة بالسل الرئوي إيجابي اللُّطَاخة وسلبي اللطاخة بعدد أيام قدره 25050 و12931 من أيام الشخص الناقل للعدوى سنويًا لتجميعة السل المعدية، على التوالي. وساهمت حالات إعادة العلاج والحالات غير المشخصة حاليًا بـ 840 840 و 34 310 يومًا من أيام الشخص الناقل للعدوى سنويًا، على التوالي. وقُدِّر إجمالي حجم تجميعة السل المعدية في منطقة غرب غوجام خلال فترة الدراسة بـ 81131 يومًا من أيام الشخص الناقل للعدوى سنويًا أو 3405 من أيام الشخص الناقل للعدوى لكل 100 000 نسمة في السنة. وبالمقارنة مع دراسة مماثلة أجريت في عام 2009 في منطقة الدراسة، أظهرت الدراسة الحالية انخفاضًا لتجميعة السل المعدية بمقدار 244279 يومًا من أيام الشخص الناقل للعدوى.
الاستنتاج: <bx/> زمن إدارة مرض السلهي أداة بسيطة وعملية يمكن أن تساعد على تقدير ومقارنة التغيرات في حجم تجميعة السل المعدية على المستوى المحلي. يمكن أيضا استخدامها كمؤشر لرصد التغيرات في أداء برنامج مكافحة السل.

Translated from English version into Arabic by Nashwa Anany, through

TRANSLATORS
WITHOUT BORDERS

采用结核病管理时间估算埃塞俄比亚地区级结核病感染人群规模

Senedu Bekele Gebreegziabher, Gunnar Aksel Bjune and Solomon Abebe Yimer

摘要
引言：估算结核病（TB）感染人群规模（infectious pool）对了解结核病控制项目的负担和监测方向至关重要。本研究采用结核病管理时间（*TB management time*）评估并比较了埃塞俄比亚阿姆哈拉州西戈杰詹姆区2009-2014年期间结核病感染人群的规模。
方法：本研究连续纳入了2013年10月-2014年10月在西戈杰詹姆区随机抽选的30家公共卫生机构中新发和复发痰涂片呈阳性及阴性的肺结核（PTB）患者。为了确定患者感染期，计算每位患者的结核病管理时间，即从开始咳嗽到进行抗结核治疗的天数。估算未确诊的结核病例数，从而计算未确诊人群的结核病管理时间。以年感染人天数（infectious person days per

year）估算研究期间当地的结核病感染人群规模。

结果： 新发痰涂片阳性和阴性肺结核患者每年分别为当地感染人群规模贡献 25 050 和 12 931 年感染人天。复发和尚未确诊病例分别贡献 8 840 和 34 310 年感染人天。研究期间，据估算西戈杰詹姆区结核感染人群规模为 81 131 年感染人天，或每年每 10 万人的感染人天数为 3 405。与 2009 年当地类似的研究结果相比，本研究显示，结核感染人群规模减少了 244 279 感染人天。

结论： 结核病管理时间是一个简单实用的工具，有助于估算和比较地方一级结核感染人群的变化，也可用作监测结核病控制项目成果的指标。

Translated from English version into Chinese by Peng Song, edited by Pin Yang

CHINESE CENTER FOR DISEASE CONTROL AND PREVENTION
NATIONAL INSTITUTE OF PARASITIC DISEASES

Appliquer *temps gestion* tuberculose pour mesurer le bassin infectieux de la tuberculose au niveau local en Ethiopie

Senedu Bekele Gebreegziabher, Gunnar Aksel Bjune et Solomon Abebe Yimer

Résumé

Contexte: Il est primordial de mesurer l'ampleur du bassin infectieux de la tuberculose (TB) pour comprendre le fardeau qu'il représente et pour surveiller l'évolution du programme de lutte contre la tuberculose. Cette étude s'est appuyée sur le *temps de gestion de la tuberculose* pour évaluer et comparer l'ampleur du bassin infectieux de la tuberculose dans la zone Godjam ouest de la région d'Amhara, en Éthiopie, entre 2009 et 2014.

Procédés: de nouveaux patients atteints de tuberculose pulmonaire (PTB) avec frottis positif et frottis négatif ainsi que des patients en retraitement, qui se sont présentés dans trente centres de santé publique choisis au hasard à Godjam Ouest, d'octobre 2013 à octobre 2014, ont participé successivement à cette étude. Afin de déterminer la période infectieuse, le *temps de gestion de la tuberculose* (nombre de jours après l'apparition de la toux jusqu'au début du traitement antituberculeux) a été calculé pour chaque catégorie de patients. Le nombre de cas de tuberculose non-diagnostiqués a été évalué et le *temps de gestion de la tuberculose* pour la catégorie non-diagnostiquée fut calculé en conséquence. L'ampleur totale du bassin infectieux de la tuberculose au cours de la période d'étude et pour la zone étudiée fut estimée en nombre annuel de jours-personnes contagieuses.

Résultats: De nouveaux cas de tuberculose pulmonaire avec frottis positif et négatif ont alimenté le bassin infectieux de la tuberculose à hauteur de 25 050 et 12 931 jours-personnes contagieuses par an. Les patients en retraitement et ceux actuellement non diagnostiqués ont représenté respectivement 8 840 et 34 310 jours-personnes contagieuses par an. L'ampleur totale du bassin infectieux de la tuberculose à Godjam ouest pendant la période étudiée a été estimée à 81 131 jours-personnes contagieuses par an ou 3 405 jours-personnes contagieuses pour 100 000 habitants par an. Comparée à une étude similaire effectuée en 2009 dans la zone étudiée, la présente étude a révélé une réduction du bassin infectieux de la tuberculose de 244 279 jours-personnes contagieuses.

Conclusions : </bx> *Le temps de gestion de la tuberculose pulmonaire* est un outil simple et

pratique pouvant permettre d'évaluer et de comparer l'évolution de l'ampleur du bassin infectieux de la tuberculose au niveau local. Il peut également être utilisé comme un indicateur pour suivre l'évolution du programme de lutte contre la tuberculose.

Translated from English version into Russian by veromarie, through

Использование *фактора времени в управлении* туберкулёзом для измерения инфекционного пула туберкулеза на местном уровне в Эфиопии

Сенеду Бекеле Гебригзиабер, Гуннар Аксель Бьюн и Соломон Абеб Йимер

Аннотация

Справочная информация: Измерение размера инфекционного пула туберкулёза (ТБ) необходимо для понимания бремени туберкулёза, а также для контроля тенденций производительности программы по управлению ТБ. Данное исследование применило фактор *времени в управлении туберкулёзом* для оценки и сравнения размера инфекционного пула заболевания в период с 2009 по 2014 годы в зоне Западного Годжама региона Амхара в Эфиопии.

Методы: В исследование были последовательно включены новые случаи лёгочного туберкулёза (ЛТБ), включая как положительные, так и отрицательные результаты бактериоскопии мазка мокроты, а также случаи повторного заболевания, отобранные по случайному принципу из числа посетителей 30 медицинских учреждений зоны Западного Годжама в период с октября 2013 года по октябрь 2014 года. С целью определения инфекционного периода по каждой категории пациентов было рассчитано *время управления туберкулёзом* (число дней с момента возникновения кашля до начала лечения заболевания). Число недиагностированных случаев туберкузёза было установлено путём оценки, и таким образом был произведён расчёт *времени управления туберкулёзом* для категории недиагностированных случаев. Общий размер инфецированного пула туберкулёза за период исследования в пределах зоны изучения оценивается как ежегодное количество инфецированных человеко-дней.

Результаты: Новые случаи лёгочного туберкулёза как при положительных, так и при отрицательных результатах бактериоскопии мазка мокроты составили соответственно 25 050 и 12 931 инфецированных человеко-дней к общему инфецированному пулу туберкулёза. Повторное лечение и недиагностированные на данный момент случаи составили соответственно 8 840 и 34 310 инфецированных человеко-дней в год. Общий размер инфецированного пула туберкулёза в зоне Западного Годжама за период исследования составил 81 131 инфецированных человеко-дней в год или 3 405 инфецированных человеко-дней на 100 000 человек населения в год. В сравнении с аналогичным исследованием, проведённым в районе изучения в 2009 году, данное исследование продемонстрировало

уменьшение размера инфецированного пула туберкулёза на 244 279 инфецированных человеко-дней.
Заключение: *Время управления туберколёзом* представляет собой простое и практическое средство, способное помочь в оценке и сравнении изменений размера инфецированного пула туберкулёза на местном уровне. Данный показатель также может быть использован в качестве индикатора при контроле изменений производительности программы по управлению ТБ.

Translated from English version into Russian by Liudmila Tomanek, through

TRANSLATORS WITHOUT BORDERS

Aplicación del concepto *tiempo de gestión de* tuberculosis para medir el grupo contagioso de tuberculosis a nivel local en Etiopía

Senedu Bekele Gebreegziabher, Gunnar Aksel Bjune y Solomon Abebe Yimer

Resumen

Antecedentes: Medir el tamaño del grupo infeccioso de tuberculosis (TB) es esencial para comprender las tendencias monetarias y de monitoreo en el desempeño del programa de control de la TB. En este estudio se aplicó el concepto de *tiempo de gestión de TB* para estimar y comparar el tamaño del grupo infeccioso de TB entre 2009 y 2014 en la zona oeste de Gojjam, en la región de Amhara, Etiopía.
Métodos: se analizaron casos nuevos de tuberculosis pulmonar (PTB), con estudios de frotis de esputo positivos y negativos, y casos de retratamiento que asistieron a 30 instalaciones de salud pública seleccionadas al azar en la zona oeste de Gojjam entre octubre de 2013 y octubre de 2014. Con el fin de determinar el período infeccioso, se calculó el *tiempo de gestión de TB* (número de días desde el inicio de la tos hasta el inicio del tratamiento antituberculoso) en cada categoría de pacientes. Se estimó el número de casos de tuberculosis no diagnosticados y a partir de ello se calculó el *tiempo de gestión de TB* para dicha categoría. Se estimó el tamaño total del grupo infeccioso de TB durante el periodo de estudio y en la zona analizada como el número anual de días de personas en estado de contagio.
Resultados: los nuevos casos con frotis positivos y frotis negativos de tuberculosis pulmonar, contribuyeron con 25 050 y 12 931 días de personas en estado de contagio por año al grupo infeccioso de TB, respectivamente. Los casos de retratamiento y los no diagnosticados contribuyeron en 8 840 y 34 310 días de contagio por año, respectivamente. El tamaño total del grupo infeccioso de TB en la zona oeste de Gojjam durante el período de estudio se estimó en 81 131 días de personas contagiosas por año o en 3 405 días de personas contagiosas cada 100 000 habitantes al año. En comparación con un estudio similar, realizado en 2009 en el área de estudio, el estudio actual mostró una reducción de 244 279 días de personas en estado de contagio del grupo infeccioso de TB.

CONCLUSIÓN: *tiempo de gestión de la TB* es una herramienta sencilla y práctica que puede ayudar a estimar y comparar los cambios en el tamaño del grupo contagioso de TB a nivel local. También puede ser utilizado como indicador para controlar los cambios en el funcionamiento de los programas de control de TB.

Translated from English version into Spanish by Ana Leticia Santos, through

TRANSLATORS
WITHOUT BORDERS
